# The Foreign Journals: Surgery

**Published:** 1839-07

**Authors:** 


### SURGERY.

### *On Varicocele, and especially on the radical Cure of that Affection.* By H. Landouzy.

Sixty persons out of every hundred are affected with varicocele. Hence the necessity of studying this disease. The term varicocele, as usually employed, includes the two terms varicocele and circocele, the first of which implies an abnormal enlargement of the veins of the scrotum; the last, of those of the spermatic cord, testicle, and epididymis. Varicocele never occurs without circocele, and, in fact, always forms a consequence of it. The term varicocele is employed in preference to that of circocele, and is understood to mean a dilatation of the veins of the scrotum and cord. The age at which it most frequently begins is from ten to thirty. Of forty-five cases, ten of which are reported by others, and thirty-five occurred in the practice of Landouzy himself,

| | | | | |
|---|---|---|---|---|
| 13 | were individuals between | 9 | and | 15 years of age, |
| 29 | . . . | 15 | . | 25 |
| 3 | . . . | 25 | . | 35. |

The anatomical conditions which dispose to the frequent occurrence of varicocele are the depending position and great length of the spermatic veins; the weakness of their parietes; the absence of valves; and, especially, the changes in respect to volume, which they are constantly undergoing. We may add to these, the pressure of a column of blood reaching from the second dorsal vertebra to the testicle, and occasional impediments offered by the inguinal canal. The disease is more frequent on the left than on the right side. It is, indeed, extremely rare on the right side, and almost never occurs only on that side. In eight out of seventeen cases, the veins of the right side were enlarged simultaneously with those of the left, but to a much less degree. It is very rarely necessary to operate on the right side. Out of 120 operations performed by M. Breschet, one only was on the right side. The chief reasons which have been assigned for the greater frequency of varicocele on the left than on the right side, are the following. 1. The right spermatic veins open into the vena cava in a direction parallel to the axis of that vessel, while the left opens into the left emulgent vein at right angles to the current of blood which flows through it. 2. The greater length of the left spermatic vein. 3. The pressure of the contents of the sigmoid flexure of the colon. With regard to this last cause, Landouzy observes that only one out of seventeen patients was affected by constipation. Amongst the occasional causes of varicocele may be mentioned, all those which either prevent the return of blood to the heart, or determine it in large quantity to the organs of generation. These need not be particularized. There seems to be no close connexion between varix and varicocele. Of fifteen cases of varicocele, one only was affected with varices, and of twenty persons who had varicose veins in the lower extremities, no single one had varicocele. The symptoms of this disease are slight at first, and its existence is usually discovered by accident. There is a feeling of weight in the testicle, perineum, and loins, and an unusual twitching in the course of the cord; the scrotum is long, pendant, and soft, and increases rapidly in volume under the influence of heat or fatigue. The patient carries the hand, at every instant, to the scrotum, in order to give it a more favorable position. If the patient is not subject to much fatigue, if he does not remain for any length of time in the erect posture, and avoids all the exciting causes of the disease, a suspensory bandage will guarantee him against further suffering. But if the disease is allowed to go on unchecked, it becomes a source of constant suffering. A short walk causes extreme fatigue, the breathing becomes hurried, the face is bathed in sweat, and expresses the deepest distress. In some cases it is impossible to assume the erect posture, without the aid of a suspensory bandage. The case of one of the most celebrated dramatic authors of France is mentioned, who had acquired the habit of composing whilst rapidly pacing his chamber. This disease entirely put a stop to his perambulations, and with them to his literary productions. He was restored by an operation performed by M. Breschet. There is one symptom which our author has never known to be absent, but which has been omitted by other writers on this subject. It is an increased perspiration of the skin of the scrotum on the side affected. This secretion is, in some cases, so abundant as to require the use of a fold of linen. The superficial veins may acquire an enormous size. One case is mentioned in which they equalled, and even surpassed, the volume of the crural vein. Some cases are quoted from Pott and Sir A. Cooper, in which the disease seems to have made a sudden attack; in these instances, Landouzy thinks that the disease had existed in a less marked form, before the acute attack commenced. The atrophy of the testicle, which took place in more than one instance in which varicocele followed an accident, is justly attributed to the accident, and not to the varicocele which was the consequence of it; nevertheless, when the disease is very much advanced, the enlarged veins compress the testicle, and cause the absorption of it. Out of fifteen cases, our author found the testicle in a more or less advanced state of atrophy in nine. The occurrence of atrophy of the testicle, as a consequence of varicocele, is established by quotations from Celsus, Callisen, and Pott. Sir A. Cooper, however, does not seem to have met with any examples. One case, mentioned by Pott, is the only one in which atrophy of both testicles took place, but Landouzy has often observed the right testicle partially atrophied in varicocele of the left side. The atrophy of the testicle is proportioned to the extent of the varicocele. Not so, however, the pain, which is often most considerable where the veins are least enlarged. This fact is attributed to an enlargement of the small veins surrounding some nervous fibres. It is to the acute pain experienced in some cases, and to the constant uneasiness present in all, that the deep melancholy common to almost all diseases of the urinary and genital organs is to be ascribed. The chief object of Landouzy's paper is to prove the superiority of M. Breschet's method of compression to all others which have been recommended. Thirteen cases are related, in all of which great relief, in the majority a perfect cure, was effected by this means. The danger, too, of inflammation of the veins is much less than when other methods are resorted to: the cure, moreover, is accomplished in a less space of time.

As a preliminary step to the performance of M. Breschet's operation for varicocele, it is necessary that the diseased veins should be considerably distended with blood, in order that none of them may escape the compressing action of the forceps; for this purpose, in summer it will be sufficient that the patient should walk for some time previously, but in winter it will be desirable that he should also take a warm bath. This precaution being taken, and the scrotum being previously shaved, the patient stands upright before the surgeon, who, if the varicocele is on the left side, grasps the right side of the scrotum with his left hand, whilst with his right he endeavours to discover the situation of the vas deferens; this is not difficult; its normal situation is at the posterior part of the cord, its form that of a cylindrical stem, equal through its whole extent, its volume that of a large crow-quill, its consistence is hard though elastic, and may be compared to that of a nerve. But the best means of assuring yourself that you hold the vas deferens, is to press it between the fingers, when the patient should feel a peculiar painful sensation, referred both to the testicle and the groin, and which can scarcely deceive either the patient or operator. Having discovered the vas deferens, the operator draws it towards the septum scroti with the thumb and forefinger, and endeavours to separate the veins from it, and to collect them towards the external part of the scrotum. This sort of subcutaneous dissection forms the only difficult part of the operation, and requires patience and attention; the separation of the vessels must be made with the greatest care, in order that no vein should remain with the vas deferens and spermatic artery. The veins being thus separated, an assistant places the first forceps on the upper part of the scrotum, transversely, and as high as possible, but far enough from the root of the penis to prevent the formation of an eschar on it; it will be found convenient to raise the penis against the abdomen. The branches of the forceps should be carried as far as possible towards the septum, excluding the vas deferens, and at the external part of the scrotum a pedicle of skin about two lines in width, and containing capillary vessels only, should be left uncompressed. As soon as the first pair of forceps is properly placed, it should be screwed tight. The second pair should then be placed, in like manner, as low down as possible, without comprising the testicle, and should be tightened in the same way. An improvement in the construction of the forceps is the introduction of a supplementary blade, which may be depressed daily, by means of a screw, so as gradually to increase the pressure without augmenting the pain. It is necessary to be careful, that this increased pressure commences towards the septum, otherwise the vas deferens might be included between the blades. In general, severe pain is felt in the scrotum and groin, immediately after the operation; but this ceases in a few hours, and no further suffering is produced. A compress dipped in cold water should be applied to the scrotum, which should be slightly elevated. The forceps may be removed, from the fifth to the seventh day. The bridle of skin on the outside of the scrotum facilitates much the cicatrization of the wound, the edges of which would otherwise be widely separated by the erections of the penis, and by the weight of the testicle.

*Journal des Connaiss. Méd.-Chir. Jan. Mar.* 1838.*

---

*A Case of Wound of the Heart.* By Dr. STEIFENSAND, of Crefeld.

WOUNDS of the heart have always been accounted extremely dangerous; and although there are examples in which death followed upon penetrating wounds of the heart, only after a lapse of some days, or even weeks, yet in general such injuries are regarded as necessarily fatal. Lately, however, doubts have arisen as to the necessarily mortal character of wounds of the heart, and cases are quoted in which cicatrices of the heart, or even foreign bodies lodged in its substance, were found after death. The following case is interesting, as it shows the continuance of the functions of the heart for some days after severe injury.

C. H., a young man, aged twenty, was stabbed on the evening of the 16th September, 1837, probably with a knife, by some person unknown. After receiving the wound, he walked about 100 paces, and then fell into the arms of his companions, exclaiming that he had been stabbed. He was carried to his house, about a mile distant, and a surgeon was immediately sent for. The wound was about an inch in length, situated to the right of and close to the sternum, between the third and fourth ribs, and running in an oblique direction downwards. It had ceased bleeding; a compress was applied, and some antiphlogistic medicine prescribed. For some days the wound appeared to be superficial, but the patient was always extremely restless, and much distressed by thirst. On the 20th, whilst at stool, there was a sudden hemorrhage from the wound, which was, for the moment, stopped by the application of a fresh compress, but returned repeatedly, so that the surgeon, who till now had not been apprehensive of danger, became alarmed, and called in Dr. Steifensand. On the afternoon of the 22d, Dr. S. found the patient lying on the back, pale, with no pulse, the extremities cold, and the respiration oppressed. The beat of the heart was audible on applying the ear to the chest, and was accompanied by a peculiar short metallic sound. Black blood flowed from the wound, and the quantity was increased by pressure on the walls of the thorax. The probe was arrested by the cartilages of the ribs, and the state of the patient did not warrant a more particular examination. The bandage was applied anew, and the patient recommended to remain as quiet as possible. The restlessness, however, still continued, and he died on the morning of the 23d.

On examining the body, the right cavity of the chest was found filled with dark fluid blood. The knife had traversed the cartilage of the fourth rib of the right side, had divided the internal mammary artery, and passed through the pericardium into the right auricle, near its junction with the ventricle. The wound of the pericardium was about three lines in extent, that of the auricle about two lines. The right lung was collapsed, and pressed upwards.

* These papers have since been published as a separate work, by M. Landouzy.

In general, wounds of the heart are not followed by immediate death. Ollivier has collected fifty-four cases of penetrating wounds of the heart, among which were twenty-nine of the right ventricle; and in these, with the exception of two cases, death ensued between the fourth and twenty-eighth days. Twelve of the cases were wounds of the left ventricle, and these, with the exception of three, which survived respectively forty-nine hours, three days, and ten days, proved immediately fatal. The greater frequency of wounds of the right ventricle depends evidently on its position, and the more sudden death in wounds of the left ventricle is owing to the difference of structure and function.

*Casper's Wochenschrift.* No. 15. 1838.

---

### *Development of Hair in the Posterior Chamber of the Eye.* By Dr. Theodore Ruete, of Göttingen.

This was the case of a man thirty years of age, by trade a tinker. On the cornea, which was in other respects quite natural, there was a slight cicatrice; the anterior chamber natural; the iris appeared unchanged in structure, but its pupillary margin was, to the greatest part of its extent, adherent to the capsule of the lens. The latter was opaque, and appeared to have several fissures in it. But the most remarkable thing was the appearance of four hairs behind the pupil, two longer and two shorter. They sprang out of the bottom of the posterior chamber, from the capsule of the lens. Besides these, a still longer hair pierced the iris to the left of the pupil, and lay stretched on the iris in the anterior chamber. This state was traced to an injury from a chip of tinned iron, which struck his eye in an incandescent state, three years ago.

*Monatsschrift für Medicin, Augenheilkunde und Chirurgie.*
*Jan. and Feb.*, 1839.

---

### *Artificial Anus made in the Groin, with success.*

An infant three days old did not present any traces of the anal opening. The raphé of the perineum extended without interruption from the scrotum to the point of the coccyx. The abdomen was tender and tympanitic, but there was no vomiting. The infant had taken the breast several times, and had passed its urine without difficulty. An incision of several lines in length was made over the supposed situation of the anus, and carried to the depth of three quarters of an inch or more, but without success. It was decided then to open the cæcum in the right iliac fossa. An incision was made near the anterior iliac spine; a small knuckle of intestine presented itself, which was replaced, and the cæcum was found without difficulty. It was opened, and several ounces of meconium immediately escaped, followed by a remarkable amelioration of the symptoms. The progress towards cure was very rapid; the alvine evacuations continued to be passed by the artificial opening, and on the eighth day after the operation the sutures were removed.

*Medizinische Zeitung für Heilkunde in Preusen.*

---

### *On the Use of Conium Maculatum in Scrofulous Ophthalmia.* By Professor Otto of Copenhagen.

Kopp, of Hanau, recommends for scrofulous ophthalmia the conium maculatum. His formula is: ℞. Ext. Conii Maculati ʒj. Aquæ Cinnamomi Spirituosæ ʒjv. Solve. Of this he gives children of two or three to four years old, and older, four drops three times a day, daily adding a drop to each dose. Blisters behind the ears, and compresses, wet with tinct. thebiaca, to the eyes were at the same time used. Professor Otto says he has cured more than thirty cases of scrofulous ophthalmia by this plan. He has, with Kopp, raised the doses as high as thirty to thirty-five drops without any bad result.

*Wochenschrift für die ges. Heilkunde. April* 6, 1839.

### *Singular Case of a Living Snake in the Stomach.* By Dr. MANDT, of St. Petersburg.

A RUSSIAN peasant, by name Abraham Isajeff, of the district of Oranienbaum, æt. 36, was suddenly roused from sleep under a tree, on the 27th of July, 1838, by the distressing sensation, as if ice-cold water was passing from his mouth to the stomach. Immediately afterwards he felt something moving in his stomach, and could even perceive the motion by the hand pressed on the part. The most distressing sensation was that of extreme coldness in the stomach; but the conviction that he had swallowed a venomous serpent rendered the poor man almost distracted. He refused to eat for fear of nourishing it, and took tobacco, brandy, emetics, purgatives, &c., partly by the advice of his friends, and partly under medical direction, in the hopes of killing the animal, or of ejecting it upwards or downwards; but all in vain. He was seen on the following day, about twenty-six hours after the accident, by Dr. Selle, who was able to detect the motion of the supposed animal by the hand, through the abdominal parietes; and who also recognized, by means of the stethoscope, sounds produced by it, viz. "a half-gurgling, half-wheezing sound, and a noise, as if a hard body struck an extended surface at irregular intervals." Dr. Selle watched the case narrowly, of all the particulars of which a minute account is given. The motions and sense of coldness continued in the region of the stomach until the 31st., when they seemed transferred to the umbilical region, and then were, for the first time, accompanied by a sense of local heaviness or weight (Schwere). This day the patient was seen by Dr. Mandt, who conducted the after-treatment. He thought he could perceive, on examining the abdomen, "a longish foreign body" in it, but was not quite certain. On the 1st of August the patient was also seen by Dr. Hassing, staff-physician-general. This was the last day the patient felt the movements, or sense of coldness in the abdomen, but the weight still continued. The man being now convinced that the snake was dead, was roused from his mental agonies, recovered his spirits and courage, and began to eat. Purgatives of calomel, castor-oil, and jalap, and clysters of infusion of valerian were persevered in which acted readily and strongly on the bowels; but without the ejection of any foreign substance. On the 7th of August the man went to see his family, promising faithfully (verspricht aber heilig) to take his purgatives, and to examine carefully all his evacuations. He showed himself again on the 9th, but could not be kept from his own home any longer. He returned, however, to the doctor's the following day, "happy and triumphant, exhibiting in a glass the expelled snake, covered with excrement," which, he said, had come away from him in two pieces; the tail-end at three o'clock, and the remainder five hours thereafter. The only parts wanting of the animal were one side of the lower jaw, the rest of the head, and a small bit of the body between the tail and abdomen. It was upwards of a foot long, and about the thickness of a full-sized man's finger, and was recognized as the common viper (*vipera berus*), by its colour, the character of the tail, &c. In several places the vertebral column was broken; and the skin everywhere exhibited marks of the digestive action, in loosened scales, &c. The internal organs were all sound.

[From the whole history of this case, so minutely detailed in the original, and from the character of the narrator and witnesses, it is hardly possible to doubt the truth of the narrative as above detailed: still it will not escape the sceptical, that the positive *proof* of the two grand facts, the swallowing and the discharge of the snake, is still wanting. It is, at least, *possible,* that the snake presented to Dr. Mandt, and of which a representation is given, may have never entered or passed from the intestinal canal of Abraham Isajeff; but we must say, in justice to this highly respectable physician, that no one who reads this interesting history, will be much disposed to doubt the facts as there related.]

*Rust's Magazin für Heilkunde*, B. liii. Heft 3. 1839.

### *Ivory Bougies.*

Charriere, surgeons' instrument maker in Paris, has exhibited to the Academy bougies and other instruments, made of flexible ivory (ivory from which the calcareous matter has been extracted). They are according to the pattern of some bougies given to him by Dr. Jüterbock, of Vienna. They serve the purpose completely of elastic gum instruments, and have the great advantage, that they may be made in a few days, whereas the preparation of caoutchouc instruments occupies several months. In a practical point of view, the ivory bougies have the advantage that, when they are dry, any desired bend or curvature may be given to them, which is retained notwithstanding their elasticity. The dryer they are on introduction the more they expand, without losing in durability and firmness.

